# Long-Term Drug Survival of TNF Inhibitor Therapy in RA Patients: A Systematic Review of European National Drug Registers

**DOI:** 10.1155/2013/764518

**Published:** 2013-10-30

**Authors:** Anamika Arora, Anadi Mahajan, Dean Spurden, Helen Boyd, Duncan Porter

**Affiliations:** ^1^HERON Health Pvt. Ltd., Chandigarh 160101, India; ^2^Pfizer Ltd., Walton Oaks, Tadworth, Surrey KT20 7NS, UK; ^3^University of Glasgow, University Avenue, Glasgow G12 8QQ, UK

## Abstract

*Objective*. The present systematic review of RA registry data was undertaken to analyse the time on treatment of licensed TNF inhibitors in patients with RA in Europe. *Methods*. English language European registry studies comparing TNF inhibitors were searched using MEDLINE, Embase, Cochrane, and WHO: ICTRP up to 16 April 2012 and proceedings of three selected conferences held between 2010 and 2012. Pooled analysis was performed to determine drug survival rates for each TNF inhibitor. *Results*. Sixteen studies met the inclusion criteria, of which 11 studies assessed biologic-naive patients and five studies included a mixed population of biologic-naive and biologic pretreated patients. The overall effectiveness of TNF inhibitors diminished with time, leading to decreased drug survival rates. Pooled drug survival rates after 60 months follow-up were 37% (infliximab), 48% (adalimumab), and 52% (etanercept). Further, in an observational study, when TNF inhibitors were used in combination with methotrexate, a longer drug survival was observed compared to TNF inhibitors alone. *Conclusion*. The findings of this systematic review indicated numerically lower drug discontinuation rates with etanercept than adalimumab, whereas infliximab had the highest rate. Further research is needed to understand the underlying mechanisms of treatment discontinuation with TNF inhibitors.

## 1. Introduction

Over the past 10 to 15 years, new treatment paradigms in rheumatoid arthritis (RA) have been developed, including early diagnosis, intensive (“treat to target”) management using disease modifying antirheumatic drugs (DMARDs) alone or in combination, and the advent of targeted biologic therapies. These new paradigms have significantly improved patient outcomes, improving quality of life and reducing joint damage.

Tumour necrosis factor (TNF) inhibitors were the first biologic drugs to be developed and have been used for >10 years. Five TNF inhibitors now have marketing authorization for the treatment of RA: adalimumab (ADA), certolizumab pegol, etanercept (ETN), golimumab, and infliximab (INF). It would be of clinical importance if one TNF inhibitor were to be more effective than the others, but there is a paucity of data about the relative effectiveness of these drugs—there have been no head-to-head randomized controlled trials comparing two or more TNF inhibitors. It is possible that short-term effectiveness is similar with all TNF inhibitors, but that long-term safety and effectiveness may differ. Furthermore, no reviews have been conducted to analyse drug survival rates of these biologic agents in real-world settings. To bridge this gap of knowledge, the present systematic review was conducted with an aim to assess the time on treatment (drug survival and reasons for discontinuation) with TNF inhibitors, from published European registry studies among patients with RA.

This review restricted its scope to data from Europe because of differences in clinical practice elsewhere (e.g., in the USA, there is much higher penetrance of TNF inhibitors in the market and a different system of reimbursement), ethnicity, or risk profile (for instance in Africa and Asia, the prevalence of endemic serious infection is much higher). The cohort size, duration of follow-up, and datasets collected vary across the long-term observational studies. Therefore, a systematic literature review methodology was adopted to investigate whether the currently available TNF inhibitors can be differentiated in terms of effectiveness or persistence on therapy.

## 2. Materials and Methods

### 2.1. Data Source

A search was conducted to identify reports from 16 registries of 13 European countries which include patients with RA and data on the time on treatment (drug survival) of licensed biologics in RA. The search strategy was designed to retrieve relevant data from the literature published up to 16 April 2012. The electronic databases searched were MEDLINE (including MEDLINE In-Process), Embase, the Cochrane Central Register of Controlled Trials (CENTRAL), and the WHO's International Clinical Trials Registry Platform Search portal. The search strategy comprised search facets for registry names, disease, and treatment. Additional information on the search strategy is provided as supplementary information (Table S1 in Supplementary Material available online at http://dx.doi.org/10.1155/2013/764518).

In addition, proceedings from three conferences held between 2010 and 2012 were hand-searched for relevant abstracts. These conferences were of the American College of Rheumatology, the European League against Rheumatism, and the British Society of Rheumatology.

### 2.2. Study Eligibility Criteria

Studies retrieved by the searches were downloaded into a systematic review database and were screened according to predefined eligibility criteria. Study protocol listing the eligibility criteria for inclusion of studies in the review is provided as supplementary information (Table S2). Initial screening of the retrieved citations was conducted independently by two reviewers on the basis of the title and the abstract. Any discrepancy between the reviewers was reconciled by a third independent reviewer. The full-text publications of all citations of potential interest were then screened for inclusion by two reviewers, with all the disagreements reconciled by a third reviewer. Registry studies assessing the licensed biologics in RA were included. However, the focus of the present publication is to assess the comparative effect of various TNF inhibitors (ETN, ADA, INF, certolizumab pegol, and golimumab) for the treatment of patients with RA.

### 2.3. Evidence Synthesis

Relevant data from all included studies were extracted in parallel by two independent reviewers, using a predefined extraction grid. Reconciliation of any differences between the two reviewers was performed by a third independent reviewer. Only one extraction dataset per study was compiled from all publications related to that study so as to avoid the error of double-counting patients in subsequent analyses. Outcomes abstracted from included citations were drug survival and retention, discontinuations due to adverse events (AEs) and inefficacy, and duration of drug survival. Drug survival rate was defined as the number of patients remaining on drug therapy at a particular time point. In cases where drug survival rates were not explicitly reported, the same was estimated from the published Kaplan-Meir survival curves using the “Engauge” software (from http://digitizer.sourceforge.net/). To assess the comparative effect of the TNF inhibitors, pooling methodology was used; pooled proportions were the average of all individual study results, weighted for the sample sizes.

The review was conducted and reported in line with Preferred Reporting Items for Systematic Reviews and Meta-Analyses (PRISMA) guidelines [17].

## 3. Results

**Figure 1:**
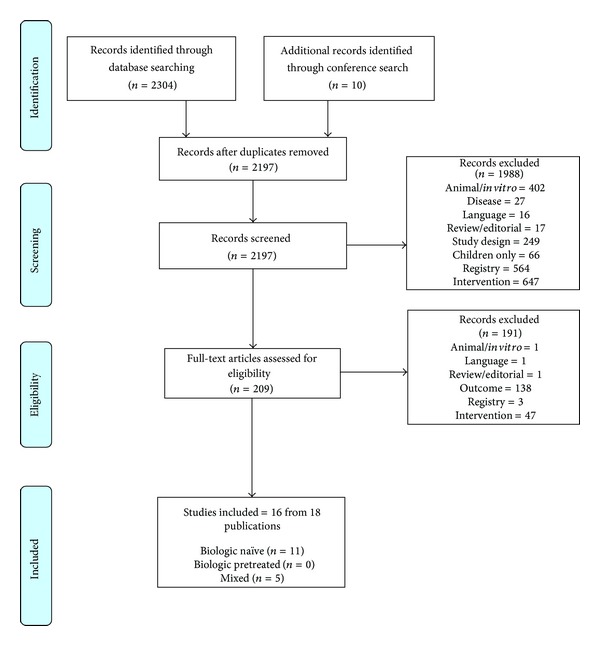
Trial flow for paper.

Figure 1 outlines the selection process for studies throughout the review, in line with PRISMA guidelines. Database searches yielded 2,304 studies and conference searching resulted in an additional 10 records. Following the screening of abstracts against inclusion/exclusion criteria, a total of 209 studies were identified and full-text citations were obtained for detailed evaluation. Of these 209 studies, 16 studies are reported in 18 publications from seven registries comparing the effect (drug survival and discontinuations) of different TNF inhibitors for the treatment of patients with RA were included (Table 1). Among these 16 included studies, drug survival rates were estimated using the Kaplan-Meir survival curves in eight studies [1–3, 7, 8, 12–14].

Of the 16 studies, 11 studies included biologic-naive patients, and the remaining five studies included a mixed population of biologic pretreated and biologic naïve patients. The studies with a mixed population predominantly included biologic-naive patients (>75%) with no subgroup analyses reported according to biologic pretreatment status. The study duration within these 16 studies ranged from 6 months [10] to 48 months [5], while the duration was not reported in nine included studies. The number of patients in these studies ranged from 66 [2] to 6739 [4]. Among the 16 included studies, 12 studies assessed the comparative effectiveness of ETN, ADA, and INF [1–8, 12–15], while the remaining four studies assessed the comparative effectiveness of ETN and INF only [9–11, 16]. None of the studies assessing certolizumab pegol and golimumab met the inclusion criteria of the review.

### 3.1. Drug Survival and Retention

**Figure 2:**
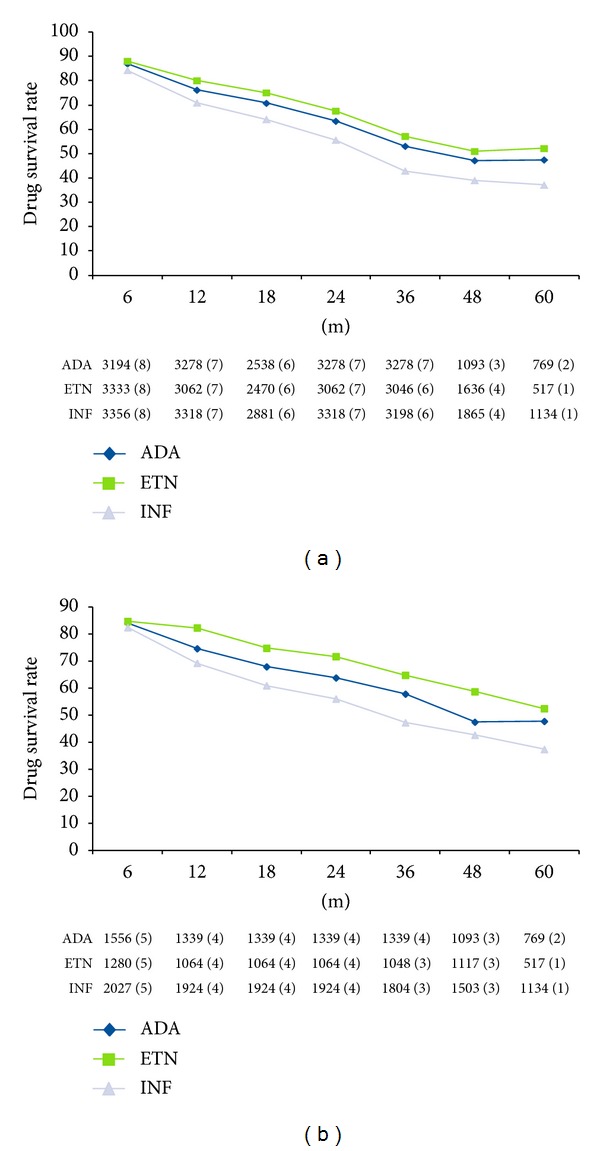
Drug survival rate (a). Overall group. (b) Biological naïve subgroup. no. of patients (no. of contributing studies) for each time point for each anti-TNF agent reported; ADA: adalimumab; ETN: etanercept; IFN: interferon.

Eleven out of twelve studies comparing ETN, ADA, and INF treatment assessed the comparative drug survival rate. Across all time points, INF was associated with numerically lowest drug survival rates in all the registries. Higher short-term (6 months) drug survival rates were reported with ADA as compared to ETN in the DREAM and SCQM registries [6, 7, 12], but the pooled drug survival rate was numerically higher with ETN compared to ADA at 6 months and beyond (Figure 2). Average drug survival rates at different time points show that approximately 7% more biologic-naïve patients persisted on ETN as compared to ADA, between 12 months and 36 months of follow-up (Table 2). In the mixed population, the difference was smaller (~4%).

Four studies assessed the comparative effect of ETN and INF only and these did not report long-term follow-up data. In the short term, little difference was observed between ETN and INF: the drug survival rates were 80.0% (ETN) versus 79.4% (INF) after 6 months and 68.9% (ETN) versus 67.8% (INF) after 12 months of treatment [9–11, 16].

In an observational study, when TNF inhibitors were used in combination with methotrexate (MTX), a benefit was observed in terms of longer drug survival compared to TNF inhibitors alone. Hyrich and colleagues conducted a study to analyze the relative effectiveness of ETN and INF as monotherapy as compared with combination of these agents with MTX. 78% of patients continued the therapy with ETN alone, while 84% of patients continued with the combination of ETN and MTX. In the INF group, after 6 months, 70% of patients continued the therapy with INF alone, while 79% of patients continued with the combination of INF and MTX [10].

Due to limited data and limited number of studies, comparison of the biologic-naive population and mixed (biologic pretreated and biologic-naive) population was not feasible.

### 3.2. Discontinuations due to Adverse Events and Inefficacy

Four studies reported the reasons for drug discontinuation; qualitative assessment of discontinuation rates comparing ETN, ADA, and INF demonstrated numerically higher discontinuation rates with INF compared to ETN and ADA for both adverse events (AEs) and inefficacy (Table 3). Due to limited data availability, comparative assessment of discontinuation rates (due to AEs or inefficacy) among studies with biologic-naive patients and studies with mixed patients was not feasible.

Three studies comparing ETN with INF only showed similar findings. After 1 year, discontinuation rates for AEs were numerically higher with INF than ETN (18.7% versus 12.6%) although discontinuation rates for inefficacy were similar (19.8% versus 18%) [9, 10, 16].

There were differences in baseline characteristics of patients among different registries—at baseline, in the study by Pan and colleagues (SCQM register), patients had DAS28 score ranging from 4.14 to 4.27 [13] but the DAS28 score ranged from 5.5 to 6.1 among patients included in the study by Strangfeld and colleagues (RABBIT register) [14]. This may partly explain differences in rates of discontinuation, with fewer patients in the SCQM cohort stopping therapy as compared to those in the RABBIT register.

### 3.3. Duration of Drug Survival

None of the included studies comparing ETN, ADA, and INF reported the duration of drug survival. Of the four studies comparing ETN with INF, only one study reported data for duration of drug survival [11]. In this study, the median duration of drug survival was higher with ETN compared to INF (45 months versus 29 months, *P* = 0.04) [11].

## 4. Discussion

Data obtained from this systematic review suggested a gradual increase in drug discontinuation over time with all TNF inhibitors mainly as a result of adverse events (AE's) and inefficacy. Drug survival with INF was numerically lower than with ETN or ADA, as a result of higher rates of AE and inefficacy. The explanations for this difference are speculative. In parts of Europe, the dose and frequency of administration are constrained by health care purchasers to the licensed dose of 3 mg/kg/8 weeks. In the USA, there is evidence that dose “creep” to 5 mg/kg/4 weeks is common and suggests that the licensed dose in Europe may not be optimal for maintaining effectiveness [18].

There were modest differences in drug survival rates with ETN compared to ADA, amounting to ~4%–7% more patients continuing on ETN over 1 year to 5 years of follow-up. There are many factors that contribute to discontinuation of therapy, including loss of effectiveness, immunogenicity, drug-related toxicity, infusion and systemic allergic reactions, and development of comorbidity [13, 19, 20]. Further, when anti-TNF agents were used in combination with MTX, a benefit was observed in terms of longer drug survival compared to TNF inhibitor alone [6].

The findings of the present systematic review are in line with the observations made in a previous network meta-analysis of updated Cochrane systematic reviews based on randomized controlled trial (RCT) data. 

The use of TNF inhibitors for the treatment of patients with RA has improved outcomes, but a significant proportion of patients discontinue therapy. Gómez-Reino and colleagues studied the rates and reasons for discontinuation of TNF inhibitors over the past decade (2000–2009). The authors observed higher discontinuation rates with these agents in recent years than a decade ago, inefficacy being the main reason for the discontinuations and also the increasing availability of alternative treatment options. The authors speculate that clinicians and patients starting biological agents nowadays might have higher expectation regarding response to therapy [21].

The present systematic review is based on observational data from the various European registers. Registry data affords some significant advantages when compared to RCTs: typically, registries contain data from large numbers of patients followed up for longer periods of time and in a real-life setting (i.e. without the exclusion criteria associated with most RCTs). However, these registry data are also associated with some limitations—in clinical practice, treatment decisions are not random but are dependent on many variables, such as disease severity, patient choice and adherence, concomitant medication, and comorbidities that may act as important confounders in analyses of efficacy and safety. The decision to stop therapy in a real-world setting due to inefficacy or AE was at the discretion of the rheumatologist and was not determined by a study protocol as in the case of RCTs. Additionally, the studies included in the present review were not designed to compare the different TNF inhibitors (none of the studies assessed statistical significance in drug survival between the TNF inhibitors), and a number of biases may have occurred. First, these three treatments became commercially available at different times (first INF, followed by ETN, and then ADA), and the patients treated with INF had a significantly higher level of disease activity and disability at the start of therapy. Universal screening for latent TB infection only became widespread once the risk of TB reactivation had been identified and might have led to higher rates of discontinuation due to TB reactivation in the early years of TNF inhibitor therapy. INF is administered via intravenous infusion, and when subcutaneous (SC) ETN and ADA became available, clinician and patient preference for this route of administration may have led to discontinuation of INF in favour of SC options. Secondly, the likelihood of patients receiving concomitant MTX therapy may vary with the choice of drug: ADA and ETN have marketing authorisation for the use of monotherapy, whereas INF is only licensed for use in combination with MTX. One study from the BSRB register published in 2006 reported that in UK, 82% of 1453 patients treated with INF received MTX as a concomitant therapy, while this proportion was only 20% in ETN group [10]. Thirdly, there is geographical variation in the use of individual TNF inhibitors which could also be related to the likelihood of developing AEs or inefficacy. There may be other unidentified confounding factors (e.g. physician preference) that may be relevant to the outcomes in question.

In spite of these facts and limitations, the results of the present systematic review provide useful insights into time and rate of survival of TNF inhibitors among the group of unselected community-based patients with severe, long-standing RA.

## 5. Conclusions

The discontinuation rates for all causes after several years of treatment were high. Approximately 50% of patients discontinue their TNF inhibitor over the first five years as a result of inefficacy or AEs. The available data indicated that the likelihood of continuing therapy was lowest with INF. There was a modest numerical increase in drug survival with ETN compared to ADA, but the clinical relevance of this difference needs to be interpreted within a global assessment of risk and benefit. Assuming equal efficacy and safety, clinicians might regard even a small increase in drug survival rate as being of significance in daily practice. Future research is needed to understand the underlying mechanisms leading to discontinuation of TNF inhibitor therapy. The discontinuation rates for all causes after several years of treatment were high. Approximately 50% of patients discontinue their TNF inhibitor over the first five years as a result of inefficacy or AEs. The available data indicated that the likelihood of continuing therapy was lowest with INF. There was a modest numerical increase in drug survival with ETN compared to ADA, but the clinical relevance of this difference needs to be interpreted within a global assessment of risk and benefit. Assuming equal efficacy and safety, clinicians might regard even a small increase in drug survival rate as being of significance in daily practice. Future research is needed to understand the underlying mechanisms leading to discontinuation of TNF inhibitor therapy.

## Supplementary Material

The electronic databases searched were MEDLINE (including MEDLINE In-Process), Embase, the Cochrane Central Register of Controlled Trials (CENTRAL), and the WHO's International Clinical Trials Registry Platform Search portal. The search strategy comprised search facets for registry names, disease, and treatment. The search strings used for searching the databases are provided in this supplementary material.

## Figures and Tables

**Table 1:** Summary of the methodological characteristics of the included studies.

Registry	Study name	Study duration	Year of entry	Study country	Number of patients included	Biologic- naive (%)
Biologic-naive						
DREAM	Flendrie et al. 2003 [1]	Maximum follow-up time for ADA, INF, and ETN was 69 months, 35 months, and 30 months, respectively.	ADA was started between April 1997 and September 2000, INF was started since January 2000, and ETN was started since February 2000	The Netherlands	230	100
SCQM	Genta et al. 2006 [2]	38 month-follow-up	Before February 2003	Switzerland	66	100
DANBIO	Hetland et al. 2010 [3]	The median (IQR) follow-up times were as follows: for ADA, 20 months (IQR: 7 months–39 months); for ETN, 21 months (IQR: 9 months–42 months); and for INF, 16 months (IQR: 5 months–36 months)	October 2000 to 31 December 2007	Denmark	2326	100
BSRBR	Hyrich et al. 2007 [4]	The mean length of follow-up per patient was 15 months (maximum 61 months)	Patients who completed minimum 6 months of follow-up by the end of April 2005	UK	6739	100
GISEA	Iannone et al. 2011 [5]	48 months	2003-2004	Italy	853	100
DREAM	Kievit et al. 2007 [6]	Mean follow-up duration was 20 months for INF cohort and 13 months for ETN and ADA cohorts	April 2003 to December 2005	The Netherlands	546	100
DREAM	Kievit et al. 2008 [7]	12 months	February 2003 to August 2007	The Netherlands	707	100
LOHREN	Marchesoni et al. 2009 [8]	36 months	1999	Italy	1064	100
BSRBR	Hyrich et al. 2006 [9]	6 months (minimum follow-up duration)	Only those patients who had reached six month follow-up prior to 1 October 2004 were included	UK	3223	100
BSRBR	Hyrich et al. 2006b [10]	6 months	Not reported	UK	2711	100
DANBIO	Østergaard et al. 2007 [11]	Not reported	Before October 2002	Denmark	417	100

Mixed (biologic pretreated and biologic-naive)						
SCQM	Finckh et al. 2006 [12]	The median follow-up time for ADA, INF, and ETN was 10.7 months (IQR: 5.8 months–12.3 months), 18.8 months (IQR: 11.5 months–28.3 months), and 23.7 months (IQR: 12.6 months–35.8 months), respectively.	Between January 1998 and September 2004	Switzerland	1198	83
SCQM	Pan et al. 2009 [13]	Not reported	January 1997 to December 2006	Switzerland	2364	77
RABBIT	Strangfeld et al. 2009 [14]	36 months	1 May 2001 to 31 December 2006	Germany	1769	81
RABBIT	Zink et al. 2006 [15]	12 months	Between May 1 2001 and December 31 2004	Germany	1458	78.3
RABBIT	Zink et al. 2005 [16]	Not reported	Patients enrolled between May 2001 and September 2003, were included and followed up to March 2004.	Germany	1523	87.9

ARTIS: Antirheumatic Therapies in Sweden; ATTRA: Anti-TNF treatment of rheumatoid arthritis; BIOBADASER: Base de Datos de Productos Biológicos de la Sociedad Española de Reumatología; BSRBR: British Society for Rheumatology Biologics Register; DANBIO: Danish Biologic Registry; DAS: disease activity score; DMARDs: disease-modifying antirheumatic drugs; DREAM: Dutch Rheumatoid Arthritis Monitoring; EMECAR: Estudio de la Morbilidad y Expresión Clínica de la Artritis Reumatoide; GISEA: Italian Group for the Study of Early Arthritis; LOHREN: Lombardy Rheumatology Network; NOR-DMARD: Norwegian Disease-modifying antirheumatic Drugs; RABBIT: rheumatoid arthritis observation of biologic therapy; RA: rheumatoid arthritis; RATIO: French Research Axed on Tolerance of Biotherapies; ROB-FIN: Register of Biological Treatment in Finland; SCQM: Swiss Clinical Quality Management in Rheumatic Diseases; SSATG: Southern Sweden Antirheumatic Therapy Group; STURE: Stockholm Tumour Necrosis Factor-*α* Follow-up Registry; TNF: tumour necrosis factor; UK: United Kingdom.

**Table 2:** Pooled drug survival rates with number of patients/studies evaluated for ETA, ADA, and INF at different time points among the included studies (12 comparative studies evidence).

Biologic pretreatment status	Intervention	6 m	12 m	18 m	24 m	36 m	48 m	60 m
Biologic-naive studies	ADA	83.9 (1556) *N* = 5	74.4 (1339) *N* = 4	67.7 (1339) *N* = 4	63.6 (1339) *N* = 4	57.6 (1339) *N* = 4	47.2 (1093) *N* = 3	47.5 (769) *N* = 2
	ETN	84.5 (1280) *N* = 5	82.1 (1064) *N* = 4	74.6 (1064) *N* = 4	71.5 (1064) *N* = 4	64.6 (1048) *N* = 3	58.5 (1117) *N* = 3	52.2 (517) *N* = 1
	INF	82.2 (2027) *N* = 5	69.0 (1924) *N* = 4	60.7 (1924) *N* = 4	55.8 (1924) *N* = 4	47.0 (1804) *N* = 3	42.4 (1503) *N* = 3	37.1 (1134) *N* = 1

Overall comparative evidence	ADA	87.0 (3194) *N* = 8	76.2 (3278) *N* = 7	70.8 (2538) *N* = 6	63.4 (3278) *N* = 7	53.1 (3278) *N* = 7	47.2 (1093) *N* = 3	47.5 (769) *N* = 2
	ETN	88.1 (3333) *N* = 8	80.1 (3062) *N* = 7	75.1 (2470) *N* = 6	67.6 (3062) *N* = 7	57.2 (3046) *N* = 6	51.0 (1636) *N* = 4	52.2 (517) *N* = 1
	INF	84.3 (3356 *N* = 8	70.9 (3318) *N* = 7	64.1 (2881) *N* = 6	55.6 (3318) *N* = 7	42.9 (3198) *N* = 6	39.0 (1865) *N* = 4	37.1 (1134) *N* = 1

ADA: adalimumab; ETN: etanercept; INF: infliximab; *N* represents the number of studies involved.

**Table 3:** Summary of discontinuation rate due to AEs and inefficacy at different time points among the included studies (12 comparative studies evidence).

Registry	Study name	Intervention	*N*	Discontinuation rate due to AEs (%)					Discontinuation rate due to inefficacy (%)				
				6 m	12 m	18 m	24 m	36 m	6 m	12 m	18 m	24 m	36 m
Biologic-naive													
DREAM	Flendrie et al. 2003 [1]	ADA	94	—	11.0	—	—	—	—	11.0	—	—	—
		ETN	14	—	7.0	—	—	—	—	7.0	—	—	—
		INF	83	—	24.0	—	—	—	—	10.0	—	—	—
LOHREN	Marchesoni et al. 2009 [8]	ADA	303	—	—	—	—	19.8	—	—	—	—	14.9
		ETN	242	—	—	—	—	11.6	—	—	—	—	12.8
		INF	519	—	—	—	—	20.4	—	—	—	—	20.0

Mixed (biologic pretreated and biologic-naive)													
SCQM	Pan et al. 2009 [13]	ADA	882	4.7	7.7	10.7	12.4	16.2	—	—	—	—	—
		ETN	887	2.8	7.4	12.5	15.4	21.1	—	—	—	—	—
		INF	595	4.7	10.0	14.8	20.3	24.7	—	—	—	—	—
RABBIT	Strangfeld et al. 2009 [14]	ADA + LEF	174	12.0	21.6	26.0	27.3	—	13.1	23.8	28.0	31.2	—
		ADA + MTX	566	12.8	18.8	23.8	25.0	—	12.8	18.5	22.4	24.6	32.4
		ETN + LEF	144	8.5	15.7	19.3	22.1	—	9.6	19.9	22.6	30.6	
		ETN + MTX	448	9.9	13.5	16.3	18.7	20.6	10.4	17.1	20.5	24.5	30.0
		INF + LEF	76	17.8	27.8	—	—	—	16.1	25	37.4	43.5	—
		INF + MTX	361	17.3	22.6	27.8	30.5	37.1	12.8	23.3	28.3	32.2	—
		ADA		8.3 (1622) *N* = 2	13.1 (1622) *N* = 2	16.9 (1622) *N* = 2	18.4 (1622) *N* = 2	16.2 (882) *N* = 1	—	—	—	—	—
Pooled discontinuation rate		ETN		5.5 (1479) *N* = 2	10.1 (1479) *N* = 2	14.3 (1479) *N* = 2	17.1 (1479) *N* = 2	20.9 (1335) *N* = 2	—	—	—	—	—
		INF		10.1 (1032) *N* = 2	15.8 (1032) *N* = 2	19.7 (956) *N* = 2	24.2 (956) *N* = 2	29.4 (956) *N* = 2	—	—	—	—	—

Overall comparative evidence													
		ADA		8.3 (1622) *N* = 2	13.0 (1716) *N* = 3	16.9 (1622) *N* = 2	18.4 (1622) *N* = 2	17.3 (1185) *N* = 2	12.8 (740) *N* = 1	18.7 (834) *N* = 2	23.8 (740) *N* = 1	26.2 (740) *N* = 1	26.4 (869) *N* = 2
Pooled discontinuation rate		ETN		5.5 (1479) *N* = 2	10.1 (1493) *N* = 3	14.3 (1479) *N* = 2	17.1 (1479) *N* = 2	19.5 (1577) *N* = 3	10.1 (592) *N* = 1	17.5 (606) *N* = 2	20.9 (592) *N* = 1	26.0 (592) *N* = 1	23.9 (690) *N* = 2
		INF		10.1 (1032) *N* = 2	16.4 (1115) *N* = 3	19.7 (956) *N* = 2	24.2 (956) *N* = 2	26.2 (1475) *N* = 3	13.3 (437) *N* = 1	21.3 (520) *N* = 2	29.7 (437) *N* = 1	34.1 (437) *N* = 1	20.0 (519) *N* = 1

ADA: adalimumab; DREAM: Dutch Rheumatoid Arthritis Monitoring; ETN: etanercept; INF: infliximab; LEF: leflunomide; LOHREN: Lombardy Rheumatology Network; M: Months; MTX: methotrexate; RABBIT: rheumatoid arthritis observation of biologic therapy; SCQM: Swiss Clinical Quality Management in Rheumatic Diseases.
