# Supplementary material for: Long-Term Drug Survival of TNF Inhibitor Therapy in RA Patients: A Systematic Review of European National Drug Registers
Source: Int J Rheumatol. 2013 Oct 30;2013:764518. doi: 10.1155/2013/764518 (PMC3838831; doi:10.1155/2013/764518)
Supplement: Supplementary file 1 — The electronic databases searched were MEDLINE (including MEDLINE In-Process), Embase, the Cochrane Central Register of Controlled Trials (CENTRAL), and the WHO's International Clinical Trials Registry Platform Search portal. The search strategy comprised search facets for registry names, disease, and treatment. The search strings used for searching the databases are provided in this supplementary material. [file 764518.f1.docx]

**Supplementary files**

Table S1: Embase.com search strategy for Embase® and MEDLINE®

| # | Search History |
| --- | --- |
| 1. | 'rheumatoid arthritis'/exp |
| 2. | (rheumatoid OR reumatoid OR revmatoid OR rheumatic OR reumatic OR revmatic) NEAR/3 (arthrit* OR artrit* OR diseas* OR condition* OR nodule*) |
| 3. | ('rheumatoid pneumoconiosis'/de OR caplan* NEXT/2 (syndrome OR disease)) |
| 4. | ('felty syndrome'/de OR felty* NEAR/1 syndrome) OR ('rheumatoid nodule'/de OR rheumat* NEXT/2 nodule) OR ('adult onset still disease'/de OR still* NEXT/1 disease) |
| 5. | #1 OR #2 OR #3 OR #4 |
| 6. | 'cohort analysis'/exp OR 'longitudinal study'/exp OR longitudinal:ab,ti |
| 7. | registr* OR register OR database* OR 'database'/syn OR 'register'/syn |
| 8. | ARTIS OR 'antirheumatic therapies in sweden' OR STURE OR 'stockholm tumor necrosis factor-a follow-up registry' |
| 9. | SSATG OR 'south swedish arthritis treatment group' |
| 10. | BSRBR OR 'british society for rheumatology biologics register' |
| 11. | 'register RABBIT' or 'german biologics register' or RABBIT |
| 12. | BIOBADASER OR 'spanish biologics register' |
| 13. | 'spanish registry of adverse events of biological therapies in rheumatic diseases' |
| 14. | EMECAR OR 'NOR DMARD' OR DANBIO OR ATTRA OR SCQM OR 'swiss clinical quality management' |
| 15. | 'hellenic registry' OR 'GISEA' OR 'LOHREN' |
| 16. | 'dutch rheumatoid arthritis monitoring' OR 'french ratio registry' OR RATIO OR DREAM |
| 17. | ‘ROB-FIN’ OR ROBFIN OR ‘Register of Biological Treatment in Finland’ |
| 18. | #6 OR #7 OR #8 OR #9 OR #10 OR #11 OR #12 OR #13 OR #14 OR #15 OR #16 OR #17 |
| 19. | 'tumor necrosis factor'/de OR 'tumor necrosis factor antibody'/de OR 'anti tumour necrosis factor':ab,ti OR 'anti tumor necrosis factor':ab,ti OR 'anti tnf':ab,ti OR ('tnfr-fc fusion' NEXT/1 protein*):ab,ti OR ('tnf receptor fusion' NEXT/1 protein*):ab,ti OR 'tnf receptor fc fusion protein':ab,ti OR 'interleukin 6 receptor':ab,ti OR 'interleukin 6r':ab,ti OR 'il 6 receptor':ab,ti OR 'il 6r':ab,ti |
| 20. | 'adalimumab'/de OR adalimumab:ab,ti OR humira:ab,ti OR 'monoclonal antibody d2e7':ab,ti OR trudexa:ab,ti |
| 21. | 'certolizumab pegol'/de OR certolizumab:ab,ti OR 'cdp 870':ab,ti OR cdp870:ab,ti OR cimzia:ab,ti OR 'pha 738144':ab,ti OR pha738144:ab,ti |
| 22. | 'etanercept'/de OR etanercept:ab,ti OR enbrel:ab,ti OR 'tnr 001':ab,ti OR tnr001:ab,ti |
| 23. | 'infliximab'/de OR infliximab:ab,ti OR avakine:ab,ti OR remicade:ab,ti |
| 24. | 'rituximab'/de OR rituximab:ab,ti OR 'idec c2b8':ab,ti OR mabthera:ab,ti OR ritux?n:ab,ti |
| 25. | 'tocilizumab'/de OR tocilizumab:ab,ti OR actemra:ab,ti OR atlizumab:ab,ti OR 'r 1569':ab,ti OR r1569:ab,ti OR roactemra:ab,ti |
| 26. | 'abatacept'/de OR abatacept:ab,ti OR orencia:ab,ti OR 'bms 188667':ab,ti OR bms188667:ab,ti OR 'ctla4 ig':ab,ti OR 'ctla4 immunoglobulin':ab,ti OR ctla4ig:ab,ti OR 'ctla-4ig':ab,ti OR 'ctla-4-ig':ab,ti OR 'recombinant interleukin 1 receptor blocking agent'/de OR ('interleukin 1 receptor' NEXT/1 (antagonist OR block*)):ab,ti |
| 27. | 'golimumab'/de OR golimumab:ab,ti OR simponi:ab,ti |
| 28. | 'anti rheumatic':ab,ti OR biologic:ab,ti |
| 29. | #19 OR #20 OR #21 OR #22 OR #23 OR #24 OR #25 OR #26 OR #27 OR #28 |
| 30. | (#5 AND #18 AND #29) |
| 31. | (#5 AND #18 AND #29) AND ([article]/lim OR [article in press]/lim OR [conference abstract]/lim OR [conference paper]/lim OR [conference review]/lim OR [editorial]/lim OR [erratum]/lim OR [letter]/lim OR [note]/lim OR [short survey]/lim) |

Table S2: Study protocol listing the eligibility criteria for inclusion/exclusion of studies in the review as per the PRISMA guidelines

| **Studies to include** | |
| --- | --- |
| Countries and registries | Major RA registries from the following European countries will be included:   - Czech Republic (ATTRA) - Denmark (DANBIO) - Finland (ROB-FIN) - France (RATIO) - Germany (RABBIT) - Greece (Hellenic Registry for Biologic Therapies) - Italy (GISEA/LOHREN) - Netherlands (DREAM) (local biologics registry of 11 hospitals) - Norway (NOR-DMARD) - Spain (BIOBADASER, EMECAR) - Switzerland (SCQM) - Sweden (ARTIS, STURE, SSATG) - UK (BSRBR) |
| Population | - Age: adults (≥16 years) - Gender: any - Race: any - Disease: RA |
| Interventions | Following interventions will be extracted:   - ABA - ADA - Certolizumab pegol - ETN - Golimumab - INF - RIT - TOC |
| Language | - English language only |
| Publication timeframe | - Database start to present date (16 April 2012) |
| **Data sources** | |
| Databases | - Embase® - MEDLINE® - MEDLINE® In-Process - Cochrane Central Register of Controlled trials (CENTRAL) - WHO: International Clinical Trials Registry Platform Search portal |
| Conference proceedings | Abstracts from three conference proceedings will be searched for the last two years:   - ACR - EULAR - BSR |

ABA: Abatacept; ACR: American College of Rheumatology; ADA: Adalimumab; ARTIS: Anti-rheumatic Therapies In Sweden; ATTRA: Anti-TNF Treatment of Rheumatoid Arthritis; BIOBADASER: Base de Datos de Productos Biológicos de la Sociedad Española de Reumatología; BSR: British Society of Rheumatology; BSRBR: British Society for Rheumatology Biologics Register; DANBIO: Danish Biologic Registry; DREAM: Dutch Rheumatoid Arthritis Monitoring; EMBASE: Excerpta Medica Database; EMECAR: Estudio de la Morbilidad y Expresión Clínica de la Artritis Reumatoide; ETN: Etanercept; EULAR: European League against Rheumatism; GISEA: Italian Group for the Study of Early Arthritis; INF: Infliximab; LOHREN: Lombardy Rheumatology Network; MEDLINE: Medical Literature Analysis and Retrieval System Online; MS: Microsoft; NOR-DMARD: Norwegian Disease-Modifying Anti-rheumatic Drugs; RABBIT: Rheumatoid Arthritis oBservation of BIologic Therapy; RA: Rheumatoid Arthritis; RATIO: French Research Axed on Tolerance of Biotherapies; RIT: Rituximab; ROB-FIN: Register of Biological Treatment in Finland; SCQM: Swiss Clinical Quality Management in Rheumatic Diseases; SSATG: Southern Sweden Anti-rheumatic Therapy Group; STURE: Stockholm Tumour Necrosis Factor-a Follow-up Registry; TOC: Tocilizumab; UK: United Kingdom; WHO: World Health Organisation
